# Prognostic value of CA125 in diffuse large B-cell lymphoma

**DOI:** 10.3389/fonc.2025.1548399

**Published:** 2025-03-07

**Authors:** Jie Zhang, Yijing Jiang, Long Chen, Linyan Xu, Bojian Ge, Xiaobing Miao, Xiaohong Xu

**Affiliations:** ^1^ Department of Oncology, Affiliated Tumor Hospital of Nantong University, Nantong, Jiangsu, China; ^2^ Department of Pathology, Affiliated Tumor Hospital of Nantong University, Nantong, Jiangsu, China; ^3^ Department of Pathophysiology, School of Medicine, Nantong University, Nantong, Jiangsu, China; ^4^ Department of Thoracic Surgery, Nantong Rici Hospital, Nantong, Jiangsu, China

**Keywords:** carbohydrate antigen 125, diffuse large B-cell lymphoma, biomarker, prognosis, overall survival, progression free survival

## Abstract

**Background:**

Carbohydrate antigen 125 (CA125) is one of the most commonly used tumor biomarker for evaluating the prognosis of solid neoplasms. It has been reported that serum CA125 is correlated with the prognosis of non-Hodgkin’s lymphoma. The objective of this study is to explore the clinical value of CA125 in diffuse large B-cell lymphoma (DLBCL).

**Methods:**

We retrospectively analyzed the clinical and pathological data in a cohort of 315 newly diagnosed patients with DLBCL. In our case, the correlations between serum CA125 and clinicopathological parameters were analyzed. Kaplan-Meier survival curve and cox proportional hazards model were applied to evaluate the prognosis. The expression of CA125 in DLBCL paraffin tissues was detected by immunohistochemistry.

**Results:**

82 patients (26%) with DLBCL had elevated serum CA125 levels at diagnosis. Elevated serum CA125 levels were associated with poor performances status, greater than or equal to 2 Extra-nodal sites, advanced Ann Arbor stage (III-IV), presence of B symptoms, presence of bulky mass, presence of effusion, intermediate/high-risk International Prognostic Index (IPI), elevated lactate dehydrogenase levels and reduced albumin levels. Patients with elevated serum CA125 levels at diagnosis had shorter progression free survival (PFS) and overall survival (OS). Multivariate analysis revealed that serum CA125, cell of origin, IPI score and albumin were independent prognostic factors for OS and PFS. In addition, the results of the immunohistochemistry indicated that none of the 82 DLBCL paraffin tissues expressed CA125 in lymphoma cells and the surrounding microenvironment cells.

**Conclusions:**

Serum CA125 detected at the initial diagnosis is a strong predictor of prognosis in patients with DLBCL.

## Introduction

Diffuse large B-cell lymphoma (DLBCL) is the most common aggressive malignant lymphoma, accounting for about 30%-40% of non-Hodgkin ‘s lymphoma (NHL) (1). DLBCL is divided into different subtypes according to clinical features, cell morphology, immunophenotype and cytogenetics, and the prognosis, diagnosis and treatment of each subtype are significantly different. Although about 60% of newly diagnosed DLBCL patients can be cured by rituximab-based immunochemotherapy, there are still some patients with relapsed and refractory (2). The widely used DLBCL prognostic model is the International Prognostic Index (IPI). However, with the rapid development of genome and transcriptome analysis technology, the prognostic value of IPI has been challenged (3). Regrettably, transcriptome sequencing is not yet universal in most developing countries and economically underdeveloped regions. It is urgent to find a simple, convenient and economical indicator to help clinicians quickly predict the prognosis of newly diagnosed DLBCL patients on the basis of IPI.

Carbohydrate antigen 125 (CA125), a repetitive peptide epitope of mucin 16 (MUC16), is a high molecular weight glycoprotein expressed by coelomocytes (4). It can not only promote the proliferation of tumor cells, but also inhibit the anti-tumor immune response. As a serum biomarker of epithelial tumors, serum CA125 is widely used in the evaluation of treatment response, prognosis evaluation and follow-up monitoring of epithelial ovarian cancer (5). In addition, serum CA125 has been found to be elevated in various epithelial-derived tumors such as lung cancer (6), breast cancer (7), pancreatic cancer (8), and gastric cancer (9), which is associated with poor prognosis. Interestingly, studies have also found that serum CA125 levels are elevated in non-epithelial malignancies, such as non-Hodgkin ‘s lymphoma (10–15). Although the prognostic value of serum CA125 in DLBCL has been reported, the conclusion is controversial (16–18). Moreover, the reason for the increase of serum CA125 levels in DLBCL is still not clear.

In order to further clarify the value of CA125 in DLBCL, we conducted a retrospective study of the largest sample at present. We analyzed the clinicopathological features of 315 patients with newly diagnosed DLBCL, aiming to evaluate the prognostic value of serum CA125 in DLBCL. Meanwhile, we performed immunohistochemistry on the pathological tissues of DLBCL to further explore the expression of CA125 in tissues.

## Materials and methods

### Patients

The study retrospectively analyzed clinical and pathological data from 315 patients initially diagnosed with DLBCL at the Affiliated Tumor Hospital of Nantong University from July 2012 to December 2021. The following variables were considered: gender, age, Eastern Cooperative Oncology Group (ECOG) performances status (PS), the number of extra-nodal sites involved, Ann Arbor stage, B symptoms, bulky mass, serosal effusion, International Prognostic Index (IPI) score, lactate dehydrogenase (LDH), albumin (ALB), cell of origin (COO) [germinal center B-cell (GCB) or non-GCB], Ki-67.

### Follow-up and end points

Patients were followed up through outpatient visits or telephone contacts until May 11, 2024, or death. Overall survival (OS) is defined as the time from the date of diagnosis to death or last follow-up, regardless of cause. Progression free survival (PFS) is defined as the time from the beginning of treatment to disease progression or recurrence, death for any cause, or the last follow-up.

### Serum CA125 measurement

Blood samples were collected from all patients at diagnosis to detect serum CA125 levels. The serum CA125 concentrations were measured by Roche E602 automated electrochemical luminescence determination immunization using the reagent kits provided by the Swiss company Roche. The reference cut-off value of serum CA125 was 35 U/mL.

### Immunohistochemistry and evaluation

Paraffin sections were heated at 70°C for an hour, dewaxed in xylene, dehydrated by gradient alcohol, antigen retrieval was performed in EDTA solution for 3 minutes, 0.3% H_2_O_2_ for blocking endogenous peroxidase, and CA125 (clone M11, MXB, China, 100μl) was incubated overnight as a primary antibody. Secondary antibodies were incubated for 30 minutes, detected using DAB, counterstained with hematoxylin, and sealed with neutral gum. Immunohistochemical staining was independently assessed by two pathologists who were unaware of the patient’s diagnosis and clinical information.

### GEO data source

The cohort of DLBCL patients, GSE181063 was obtained from the Gene Expression Omnibus (GEO) (https://www.ncbi.nlm.nih.gov/geo/) database to assess the relationship between the levels of CA125 mRNA (MUC16) and prognosis. The data set met the following criteria (1): CA125 mRNA levels were detected; (2) Tissue samples; (3) Follow-up information contained survival time and survival status.

### Statistical analysis

Statistical analyses were performed by Statistical Package for the Social Sciences (SPSS) 25.0. The survival curves were performed by GraphPad Prism 10.0. The relationship between serum CA125 and clinicopathologic factors were analyzed using two-sided χ^2^ test. The Cox proportional hazards model was used to analyze univariate and multivariate associations between prognostic factors and OS and PFS. Using Omnibus test verified the reliability of the model. Kaplan-Meier method was used to explore the effect of serum CA125 on survival, the log-rank test was used to make the comparison. Statistical significance was defined as *P* < 0.05 (two-sided).

## Results

### Relationship between serum CA125 and clinicopathological characteristics

Characteristics of the 315 newly diagnosed DLBCL patients are shown in **Table 1**. The median age at diagnosis of patients was 67 years (range, 11-92 years), 159 (50.5%) patients were male, and 210 (66.7%) patients were older than 60 years. The frequency of elevated serum CA125 levels in newly diagnosed patients was 26% (82/315). Higher levels of serum CA125 were significantly associated with poor ECOG PS (*P* < 0.001), greater than or equal to 2 Extra-nodal sites (*P* < 0.001), advanced Ann Arbor stage (III-IV) (*P* < 0.001), presence of B symptoms (*P* = 0.001), presence of bulky mass (*P* = 0.005), presence of effusion (*P* < 0.001), intermediate/high-risk IPI (3-5 scores) (*P* < 0.001), elevated LDH levels (*P* < 0.001), reduced ALB levels (*P* < 0.001).

**Table 1:** Relationship between serum CA125 and clinicopathological characteristics of the patients with newly diagnosed DLBCL (n =315).

Variables	All patients (n=315)	Serum CA125		*P* value
		Low (n=233)	High (n=82)	
Gender				0.150
Male	159(50.5)	112(48.1)	47(57.3)	
Female	156(49.5)	121(51.9)	35(42.7)	
Age, y				0.650
> 60	210(66.7)	157(67.4)	53(64.6)	
≤ 60	105(33.3)	76(32.6)	29(35.4)	
ECOG PS				< 0.001
≥ 2	58(18.4)	32(13.7)	26(31.7)	
< 2	257(81.6)	201(86.3)	56(68.3)	
Extra-nodal sites				< 0.001
≥ 2	86(27.3)	48(20.6)	38(46.3)	
< 2	229(72.7)	185(79.4)	44(53.7)	
Ann Arbor stage				< 0.001
I-II	141(44.8)	122(52.4)	19(23.2)	
III-IV	174(55.2)	111(47.6)	63(76.8)	
B symptoms				0.001
Yes	39(12.4)	20(8.6)	19(23.2)	
No	276(87.6)	213(91.4)	63(76.8)	
Bulky mass				0.005
Yes	47(14.9)	27(11.6)	20(24.4)	
No	268(85.1)	206(88.4)	62(75.6)	
Effusion				< 0.001
Yes	62(19.7)	26(11.2)	36(43.9)	
No	253(80.3)	207(88.8)	46(56.1)	
IPI score				< 0.001
0-2	168(53.3)	145(62.2)	23(28.0)	
3-5	147(46.7)	88(37.8)	59(72.0)	
LDH				< 0.001
> ULN	210(66.7)	139(59.7)	71(86.6)	
normal	105(33.3)	94(40.3)	11(13.4)	
ALB				< 0.001
normal	263(83.5)	213(91.4)	50(61.0)	
< ULN	52(16.5)	20(8.6)	32(39.0)	
COO (Han’s)				0.439
GCB	71(22.5)	50(21.5)	21(25.6)	
non-GCB	244(77.5)	183(78.5)	61(74.4)	
Ki-67				0.713
≥ 75%	205(65.1)	153(65.7)	52(63.4)	
< 75%	110(34.9)	80(34.3)	30(36.6)	

Unless otherwise indicated, data are n (%).

CA125, Carbohydrate Antigen 125; ECOG, Eastern Cooperative Oncology Group; PS, Performances Status; IPI, International Prognostic Index; LDH, lactate dehydrogenase; ALB, albumin; COO, cell of origin; GCB, germinal center B-cell.

### Prognostic significance of serum CA125

**Figure 1:**
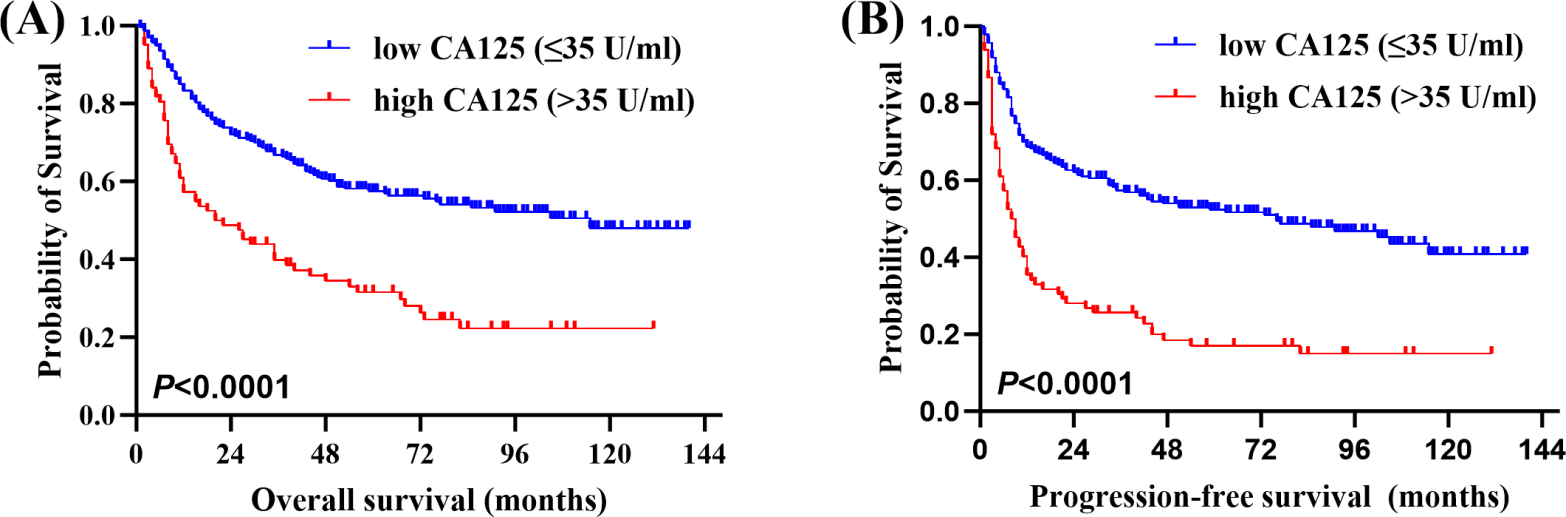
Correlation between serum carbohydrate antigen 125 (CA125) levels and survival. **(A)** Overall survival (OS) and **(B)** Progression-free survival (PFS) of newly diagnosed diffuse large B-cell lymphoma (DLBCL) patients by pretreatment serum CA125 levels.

**Figure 2:**
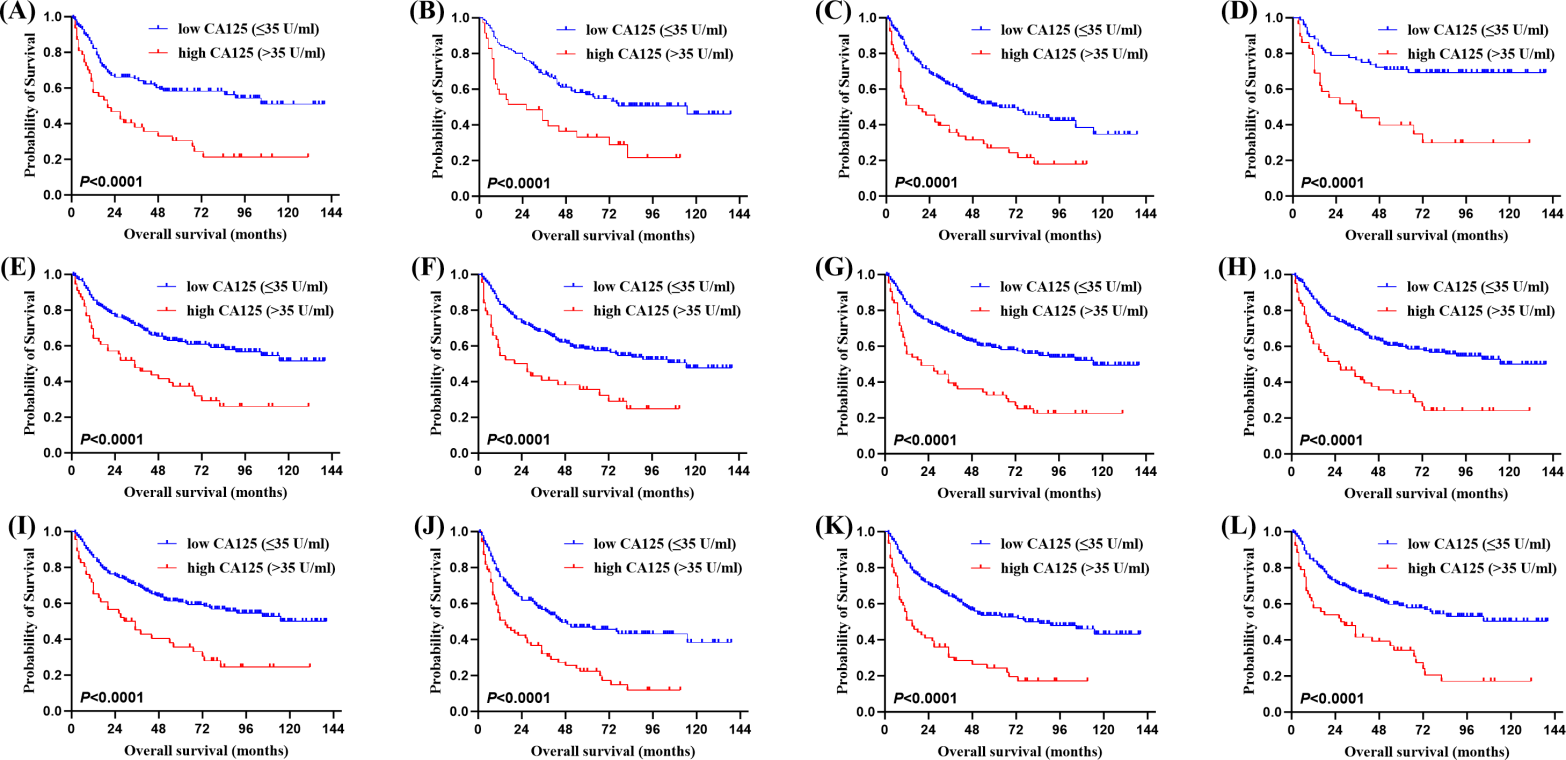
Kaplan-Meier survival curves of patients with DLBCL. OS of different serum CA125 levels in **(A)** the male group, **(B)** the female group, **(C)** the age > 60 group, **(D)** the age ≤ 60 group, **(E)** the Eastern Cooperative Oncology Group performance status (ECOG PS) < 2 group, **(F)** the extra-nodal sites < 2 group, **(G)** the absence of B symptoms group, **(H)** the absence of bulky mass group, **(I)** the absence of effusion group, **(J)** the lactate dehydrogenase (LDH) > ULN group, **(K)** the cell of origin (COO) (Han’s) non-germinal center B-cell (non-GCB) group, **(L)** the Ki-67 ≥ 75% group.

**Figure 3:**
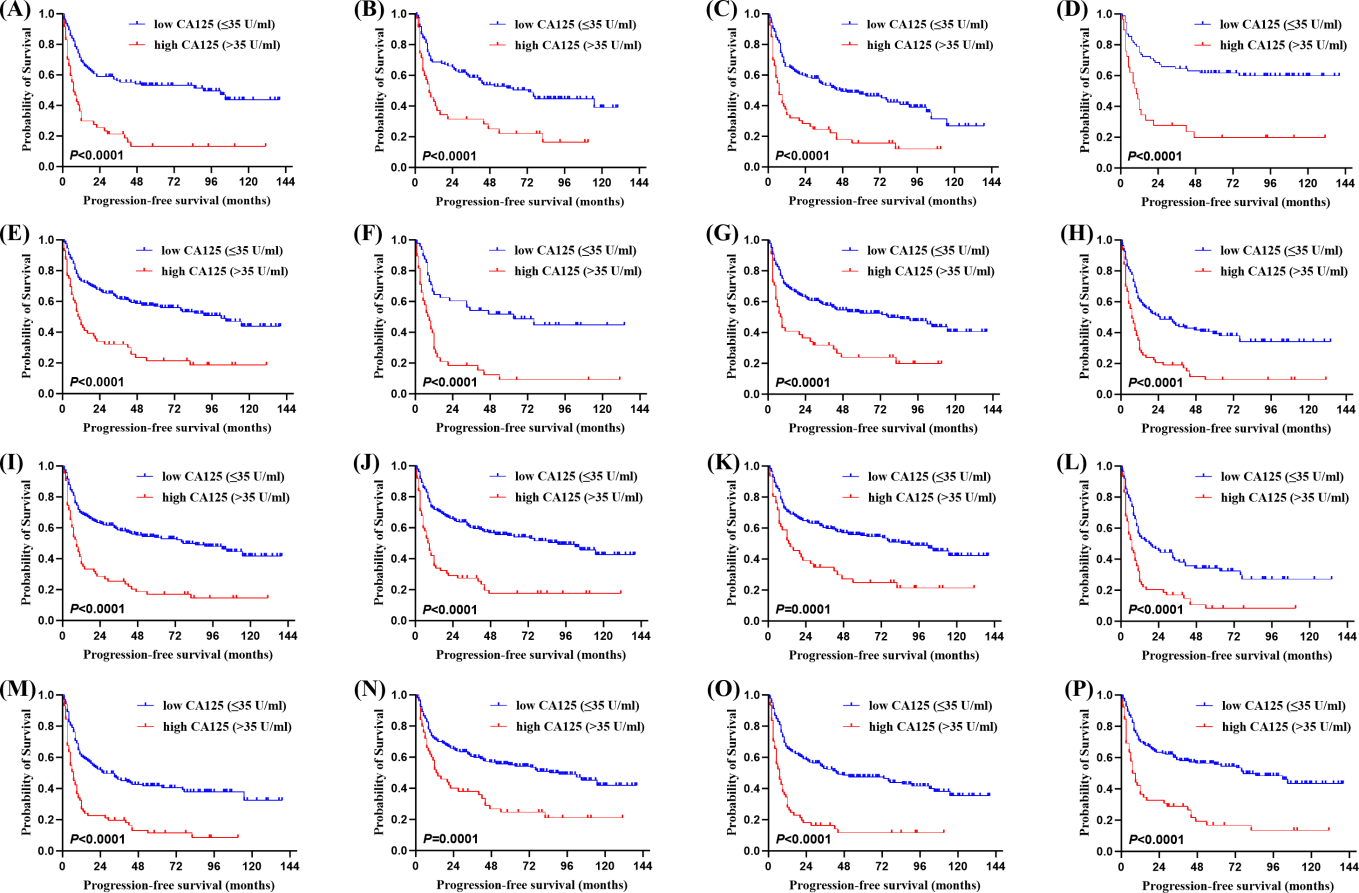
Kaplan-Meier survival curves of patients with DLBCL. PFS of different serum CA125 levels in **(A)** the male group, **(B)** the female group, **(C)** the age > 60 group, **(D)** the age ≤ 60 group, **(E)** the ECOG PS < 2 group, **(F)** the extra-nodal sites ≥ 2 group, **(G)** the extra-nodal sites < 2 group, **(H)** the Ann Arbor stage III-IV group, **(I)** the absence of B symptoms group, **(J)** the absence of bulky mass group, **(K)** the absence of effusion group, **(L)** the International Prognostic Index (IPI) score 3-5 group, **(M)** the LDH > ULN group, **(N)** the albumin (ALB) normal group, **(O)** the COO (Han’s) non-GCB group, **(P)** the Ki-67 ≥ 75% group.

Of the 315 newly diagnosed patients, the associations between serum CA125 and survival were analyzed. Patients with pretreatment elevated serum CA125 levels had shorter OS and PFS than normal levels (**Figure 1**). In order to further explore the prognostic value of serum CA125, subgroup analysis was performed. Serum CA125 in the male, female, age > 60, age ≤ 60, ECOG PS < 2, extra-nodal sites < 2, absence of B symptoms, absence of bulky mass, absence of effusion, LDH > ULN, non-GCB, Ki-67 ≥ 75% groups were able to re-stratify significantly the OS of patients (*P* < 0.0001; **Figure 2**). It could also re-stratify the OS in the extra-nodal sites ≥ 2, Ann Arbor stage I-II, Ann Arbor stage III-IV, IPI score 3-5, ALB normal, GCB, Ki-67 < 75% groups (*P* < 0.05; **Supplementary Figure S1**). Similarly, in the male, female, age > 60, age ≤ 60, ECOG PS < 2, extra-nodal sites ≥ 2, extra-nodal sites < 2, Ann Arbor stage III-IV, absence of B symptoms, absence of bulky mass, absence of effusion, IPI score 3-5, LDH > ULN, ALB normal, non-GCB, Ki-67 ≥ 75% groups, the PFS of patients were re-stratified significantly by serum CA125 (*P* < 0.0001; **Figure 3**). The PFS also could be re-stratified in the ECOG PS ≥ 2, Ann Arbor stage I-II, presence of B symptoms, presence of effusion, IPI score 0-2, ALB < ULN, GCB, Ki-67 < 75% groups by serum CA125 (*P* < 0.05; **Supplementary Figure S3**). However, serum CA125 could not re-stratify patients’ OS in the ECOG PS ≥ 2, presence of B symptoms, presence of bulky mass, presence of effusion, IPI score 0-2, LDH normal, ALB < ULN groups (**Supplementary Figure S2**) and PFS in the presence of bulky mass and LDH normal groups (**Supplementary Figure S4**).

### Univariable and multivariable analysis of OS and PFS in all patients

Covariates such as gender, bulky mass, COO, B symptoms, effusion, IPI score, ALB, serum CA125, Ki-67 were analyzed by Cox regression analysis (**Table 2**). Univariable analysis showed that bulky mass, COO, B symptoms, effusion, IPI score, ALB, serum CA125 were prognostic risk factors, and the difference was statistically significant (*P* < 0.05). Multivariate Cox regression analysis further identified non-GCB COO, IPI score 3-5, low ALB < ULN, and elevated serum CA125 levels as independent predictors of poorer OS and PFS.

**Table 2:** Univariate and multivariate analysis of prognostic factors for OS and PFS in patients with DLBCL.

Characteristics	Variable	Reference	OS				PFS			
			Univariate		Multivariate		Univariate		Multivariate	
			HR (95% CI)	*P* value	HR (95% CI)	*P* value	HR (95% CI)	*P* value	HR (95% CI)	*P* value
Gender	Female	Male	0.923 (0.680-1.252)	0.607			0.920 (0.690-1.225)	0.567		
Bulky mass	≥ 7.5cm	< 7.5cm	1.883 (1.285-2.760)	0.001	1.330 (0.895-1.977)	0.158	1.777 (1.238-2.549)	0.002	1.198 (0.822-1.747)	0.347
Cell of origin	Non-GCB	GCB	1.820 (1.197-2.767)	0.005	1.900 (1.237-2.918)	0.003	1.813 (1.226-2.681)	0.003	1.883 (1.263-2.807)	0.002
B symptoms	Yes	No	1.627 (1.060-2.497)	0.026	0.920 (0.586-1.443)	0.715	1.511 (1.003-2.276)	0.048	0.972 (0.638-1.481)	0.895
Effusion	Yes	No	2.171 (1.534-3.073)	< 0.001	1.111 (0.743-1.661)	0.607	2.495 (1.803-3.453)	< 0.001	1.349 (0.933-1.950)	0.112
IPI score	3-5	0-2	2.961 (2.149-4.080)	< 0.001	2.148 (1.508-3.059)	< 0.001	2.690 (1.996-3.625)	< 0.001	1.852 (1.328-2.582)	< 0.001
ALB	< ULN	normal	3.072 (2.148-4.392)	< 0.001	1.797 (1.212-2.663)	0.004	3.075 (2.186-4.327)	<0.001	1.745 (1.199-2.539)	0.004
CA125	> ULN	≤ ULN	2.277 (1.656-3.132)	< 0.001	1.681 (1.167-2.421)	0.005	2.556 (1.891-3.455)	<0.001	1.900 (1.355-2.665)	< 0.001
Ki-67	≥ 75%	< 75%	0.941 (0.685-1.292)	0.707			0.876 (0.651-1.179)	0.383		

OS, overall survival; PFS, progression-free survival; HR, hazard ratio; CI, confidence interval.

### Expression of CA125 on patient tissues

**Figure 4:**
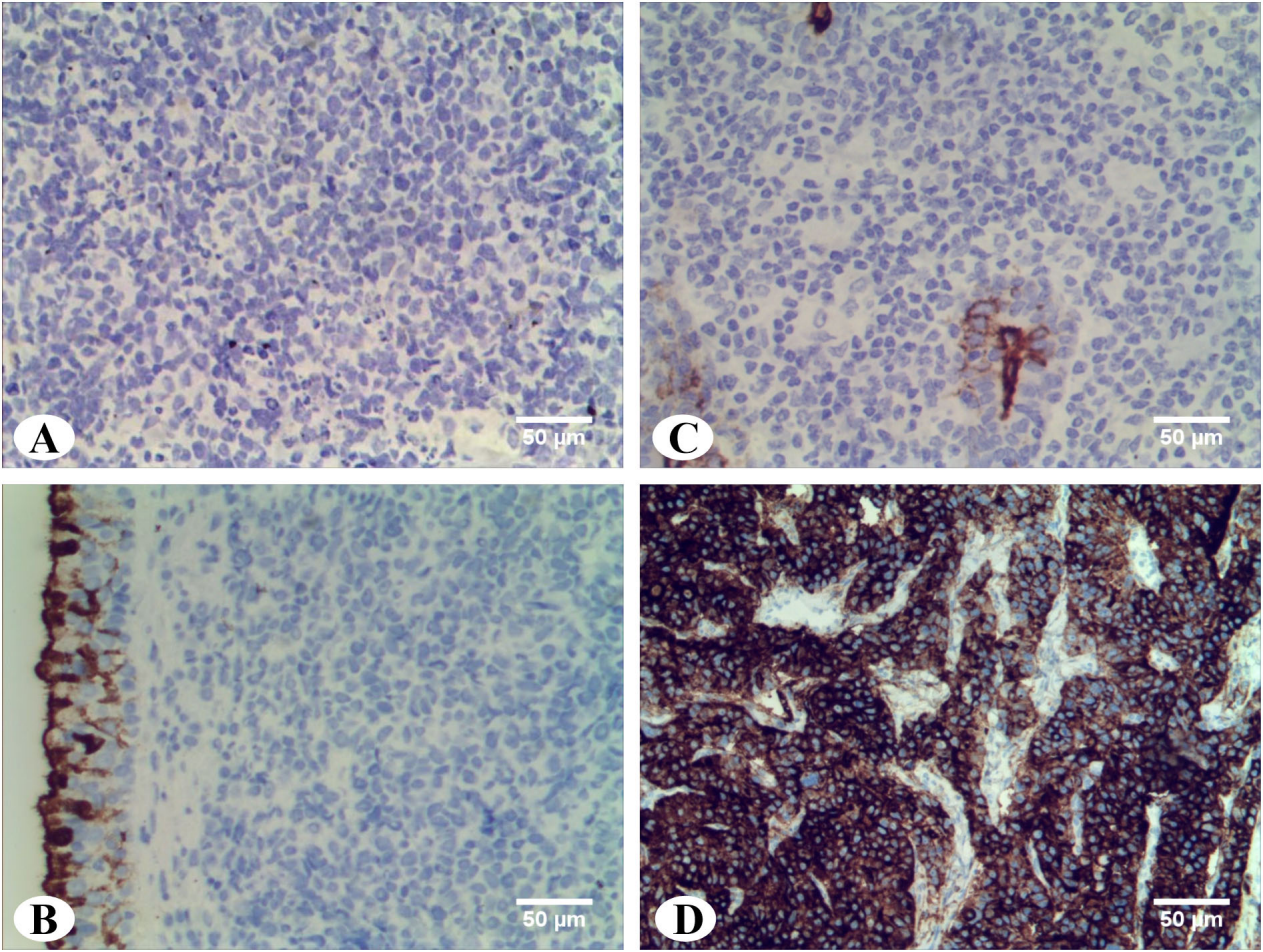
Immunohistochemistry staining of CA125 in DLBCL tissues. **(A)** Abdominal DLBCL: CA125 staining were negative in tumor cells and surrounding microenvironment cells (magnification ×20). **(B)** Parotid gland DLBCL: CA125 staining were negative in tumor cells and surrounding microenvironment cells. Residual duct epithelium cells were positive for internal control (magnification ×20). **(C)** Nasopharyngeal DLBCL: CA125 staining were negative in tumor cells and surrounding microenvironment cells. Pseudostratified columnar ciliated epithelium cells were positive for internal control (magnification ×20). **(D)** Ovarian tissue: Positive control of CA125 (magnification ×20).

In order to detect the expression of CA125 protein in DLBCL tissues, immunohistochemical staining was performed on paraffin-embedded sections from 82 DLBCL cases with elevated serum CA125 levels. While CA125 positivity was observed in epithelial cells within some surgical specimens (**Figures 4B, C**), all tumor cells and surrounding microenvironment cells were negative for CA125 staining (**Figure 4A**) compared with the positive control (**Figure 4D**).

## Discussion

The utility of CA125 has been extensively investigated in various malignancies beyond epithelial ovarian cancer. Several clinical studies have reported elevated serum CA125 levels in some patients with NHL, but the prognostic significance of this biomarker, particularly in DLBCL, remains unclear. This study represents the largest cohort clinical study to date assessing the prognostic value of CA125 in DLBCL. Our findings indicate that 26% of DLBCL patients exhibited elevated serum CA125 levels at diagnosis. Notably, elevated serum CA125 is independent prognostic risk factor for OS and PFS.

In our cohort, elevated serum CA125 levels at diagnosis were found to be relevant with poor ECOG PS, greater than or equal to 2 Extra-nodal sites, advanced Ann Arbor stage (III-IV), presence of effusion, intermediate/high-risk IPI (3-5 scores), elevated LDH levels, reduced ALB levels. These findings are consistent with a recent study on DLBCL (18). Moreover, we found that the presence of B symptoms and bulky mass were associated with the elevated levels of serum CA125 in DLBCL. This observation contrasts with that of another DLBCL cohort (17), potentially attributable to differences in sample size. However, the similar result has been reported in the study of NHL (12). Collectively, our findings confirm that elevated serum CA125 is associated with more adverse prognostic factors in DLBCL.

The prognostic value of serum CA125 in lymphoma has remained unclear over the past two decades. Four single-center retrospective studies from different regions showed that NHL patients with elevated serum CA125 levels at diagnosis had poorer OS (12–15). However, an earlier study concluded that elevated serum CA125 levels at diagnosis only predicted prognosis in patients with low-grade NHL (10). Conversely, another study found no prognostic value of serum CA125 in NHL or Hodgkin lymphoma (HL). The inconsistent findings across these studies underscore the need for further research to elucidate the clinical utility of serum CA125 as a prognostic biomarker in DLBCL (19). In a study of 42 DLBCL cases, elevated serum CA125 levels at diagnosis correlated with PFS, but not OS. However, OS became statistically significant after sex/age adjusted (17). Another study of 181 cases suggested that elevated serum CA125 at diagnosis was associated with both PFS and OS, but was an independent prognostic risk factor only for PFS (18). These discrepant results may be attributed to differences in number of cases, duration of follow-up and treatment regimens. Our retrospective analysis of 315 patients with DLBCL revealed that elevated serum CA125 at diagnosis was an independent prognostic risk factor for PFS but also for OS. It is noteworthy that this is the first report on the prognostic significance of serum CA125 at diagnosis across DLBCL subgroups with diverse clinicopathological characteristics, indicating that serum CA125 can further stratify prognosis within these subgroups.

CA125 is a high molecular weight transmembrane glycoprotein encoded by the MUC16 gene that is normally expressed by epithelial cells. Under pathological conditions, the extracellular domain of CA125-positive cells undergoes phosphorylation in the intracellular region and subsequent proteolytic cleavage, leading to its release from the cell surface and formation of serum CA125 (20). However, the mechanism of origin of serum CA125 in lymphomas has not been clarified. The secretion of lymphoma cells provides a possible explanation for the increased serum CA125 of DLBCL patients. It has been reported that the pleural effusion of an anaplastic large cell lymphoma patient with elevated serum CA125 and serum interleukin (IL) -6 levels was cultured *in vitro* and found that IL-6 can stimulate the growth of lymphoma cells and promote the release of CA125 (21). In addition, immunohistochemistry was performed in a DLBCL patient with elevated serum CA125 levels and CA125 was positive in the cytoplasm of lymphoma cells (22). Our immunohistochemical results of 82 cases show that the expression of CA125 is not found in lymphoma cells of DLBCL patients with elevated serum CA125 levels. We speculate that this negative result may be related to tumor heterogeneity. The alternative one believed that serum CA125 in lymphoma is derived from human mesothelial cells. *In vitro* experiments have shown that human mesothelial cells release CA125 from the cell surface, and IL-1β, tumor necrosis factor-α, and lipopolysaccharide can also promote the release of CA125 (23). Previous studies have reported that the expression of CA125 is positive in the mesothelial cells of the pericardium, thoracoabdominal membrane in patients with advanced lymphoma with elevated serum CA125 levels (24, 25). Xu et al. further demonstrated the relationship between serum CA125 and dropsy of serous cavity in the initial diagnosis and course of DLBCL (18). Similarly, our experimental results show that the increase of serum CA125 at the initial diagnosis is significantly associated with dropsy of serous cavity. Interestingly, there are still some patients with elevated serum CA125 levels without serous effusion, which indirectly indicates that serous cavity mesothelial cells are not the only source of serum CA125 in DLBCL. In addition, we found positive expression of CA125 in ciliated columnar epithelial cells of nasopharyngeal DLBCL and ductal cells of parotid DLBCL, respectively. Whether this is another possible source of serum CA125 remains to be further explored. In the future, we plan to construct the DLBCL mouse xenograft model to explore the source of CA125 and its potential mechanism.

It is noteworthy that the relationship between CA125 mRNA and the prognosis of DLBCL has not been reported. Based on the GEO database, we found that although CA125 mRNA is lowly expressed in lymphoma cells, elevated CA125 mRNA is still an adverse prognostic factor for OS (**Supplementary Figure S5**).

Regrettably, this is a retrospective study, and the limitation clinical data of patients with DLBCL may be caused by review bias and selection bias. Furthermore, whether CA125 has different prognostic significance in different subtypes of DLBCL such as NOS-DLBCL remains to be further studied.

In conclusion, our comprehensive investigation disclosed the clinical significance of CA125 in DLBCL. Serum CA125 at diagnosis can be used as a cost-effective, simple and non-invasive biomarker to evaluate the prognosis of patients with DLBCL. Our results validate a significant prognostic molecule, which provides clinicians with a preferred prognostic predictor for DLBCL patients who lack precision classification based on next-generation sequencing.

## Data Availability

The original contributions presented in the study are included in the article/**Supplementary Material**. Further inquiries can be directed to the corresponding authors.
