# Supplementary material for: Prognostic value of CA125 in diffuse large B-cell lymphoma
Source: Front Oncol. 2025 Mar 7;15:1548399. doi: 10.3389/fonc.2025.1548399 (PMC11925774; doi:10.3389/fonc.2025.1548399)
Supplement: Supplementary file 1 [file DataSheet1.docx]

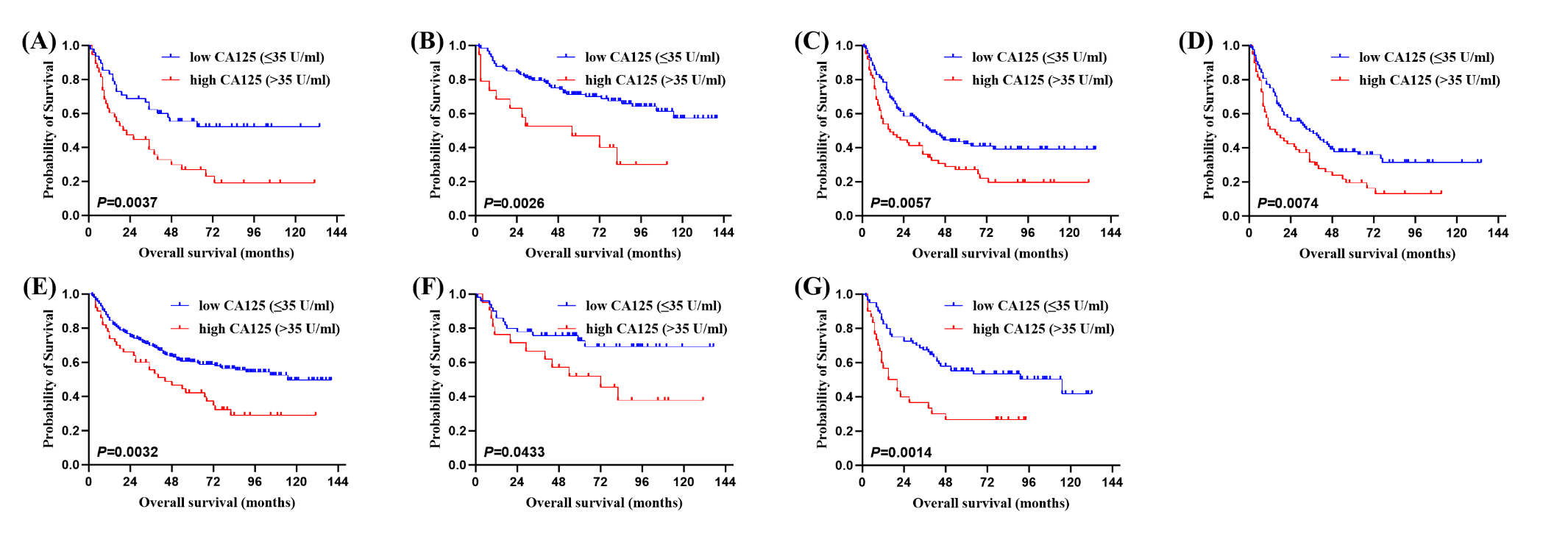


**Figure S1.** Kaplan-Meier survival curves of patients with DLBCL. OS of different serum CA125 levels in (A) the extra-nodal sites ≥ 2 group, (B) the Ann Arbor stage Ⅰ-Ⅱ group, (C) the Ann Arbor stage Ⅲ-Ⅳ group, (D) the IPI score 3-5 group, (E) the ALB normal group, (F) the COO(Han’s) GCB group, (G) the Ki-67 < 75% group.


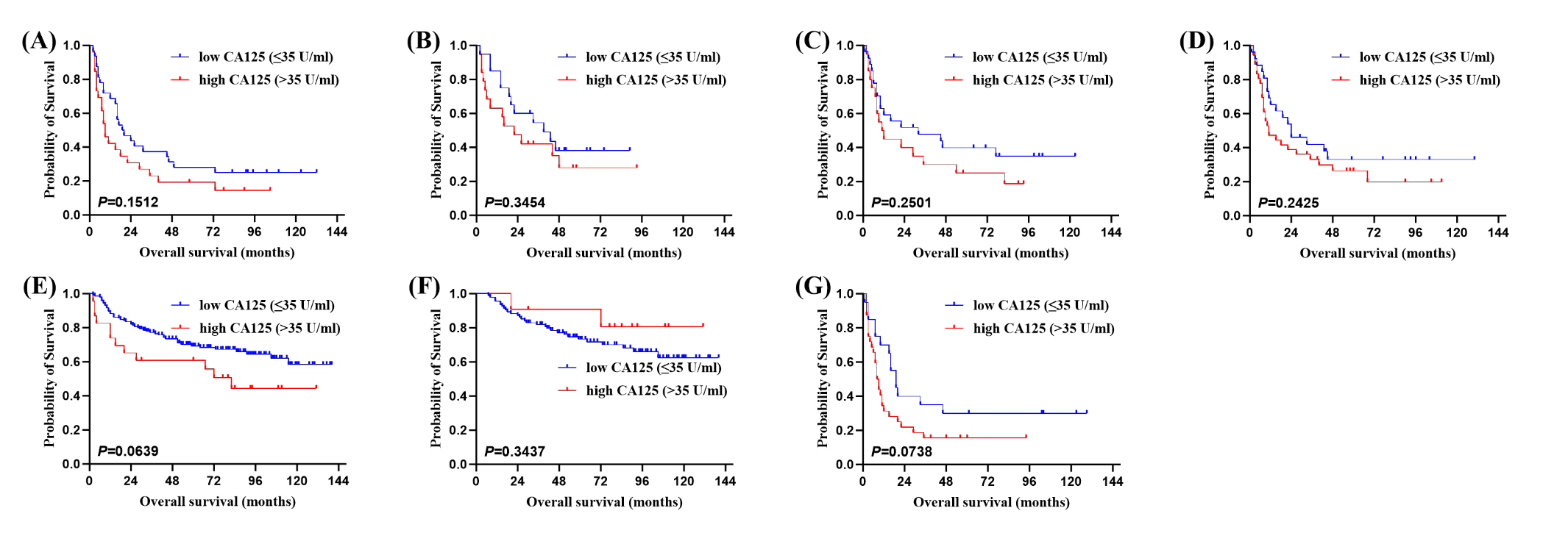
 **Figure S2.** Kaplan-Meier survival curves of patients with DLBCL. OS of different serum CA125 levels in (A) the ECOG PS ≥ 2 group, (B) the presence of B symptoms group, (C) the presence of bulky mass group, (D) the presence of effusion group, (E) the IPI score 0-2 group, (F) the LDH normal group, (G) the ALB < ULN group.


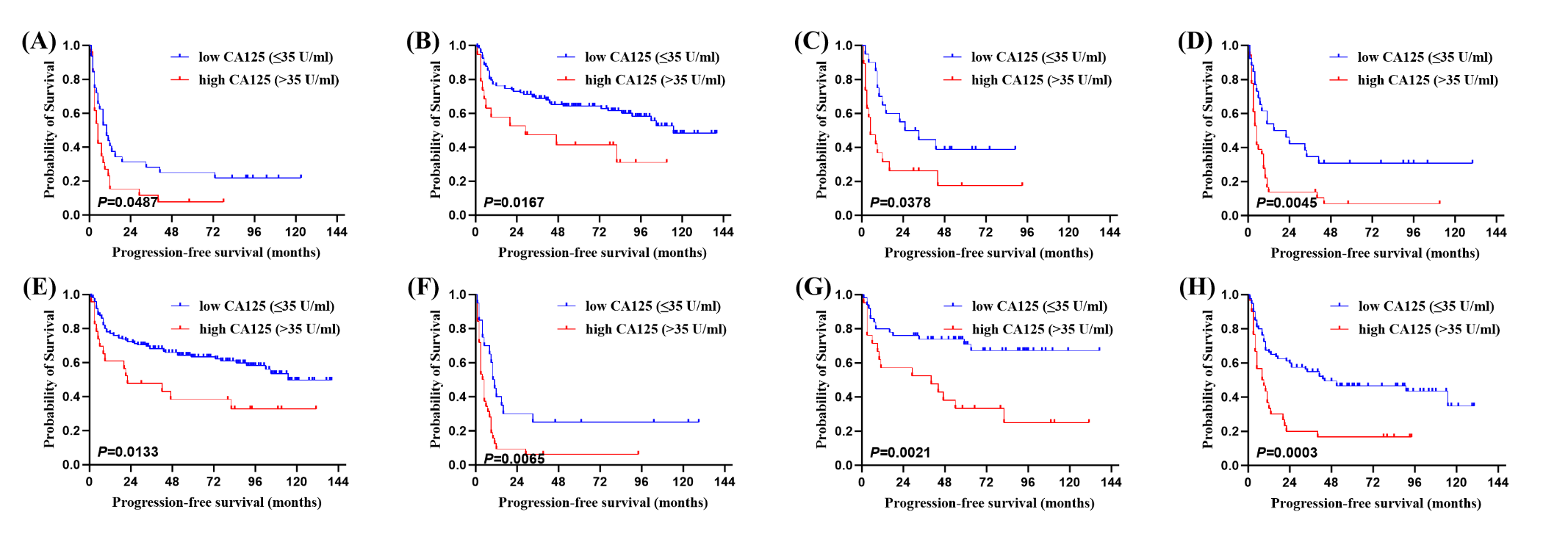
 **Figure S3.** Kaplan-Meier survival curves of patients with DLBCL. PFS of different serum CA125 levels in (A) the ECOG PS ≥ 2 group, (B) the Ann Arbor stage Ⅰ-Ⅱ group, (C) the presence of B symptoms group, (D) the presence of effusion group, (E) the IPI score 0-2 group, (F) the ALB < ULN group, (G) the COO (Han’s) GCB group, (H) the Ki-67 < 75% group.


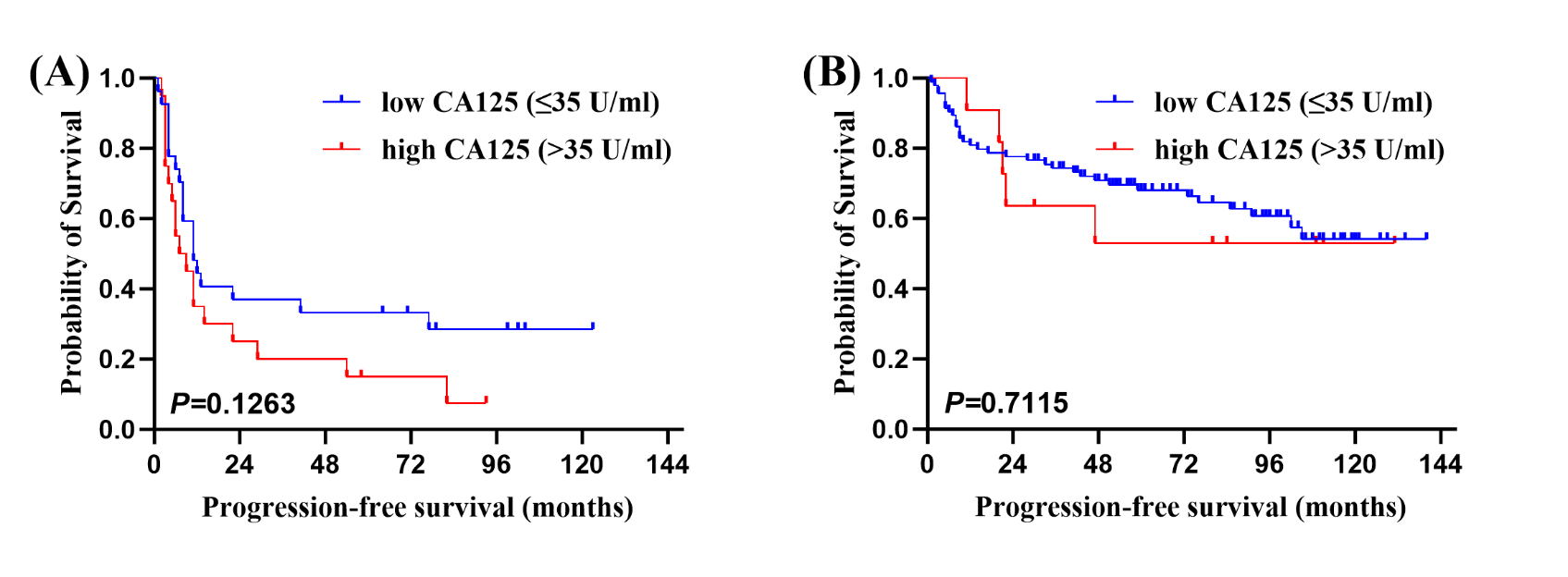


**Figure S4.** Kaplan-Meier survival curves of patients with DLBCL. PFS of different serum CA125 levels in (A) the presence of bulky mass group, (B) the LDH normal group.


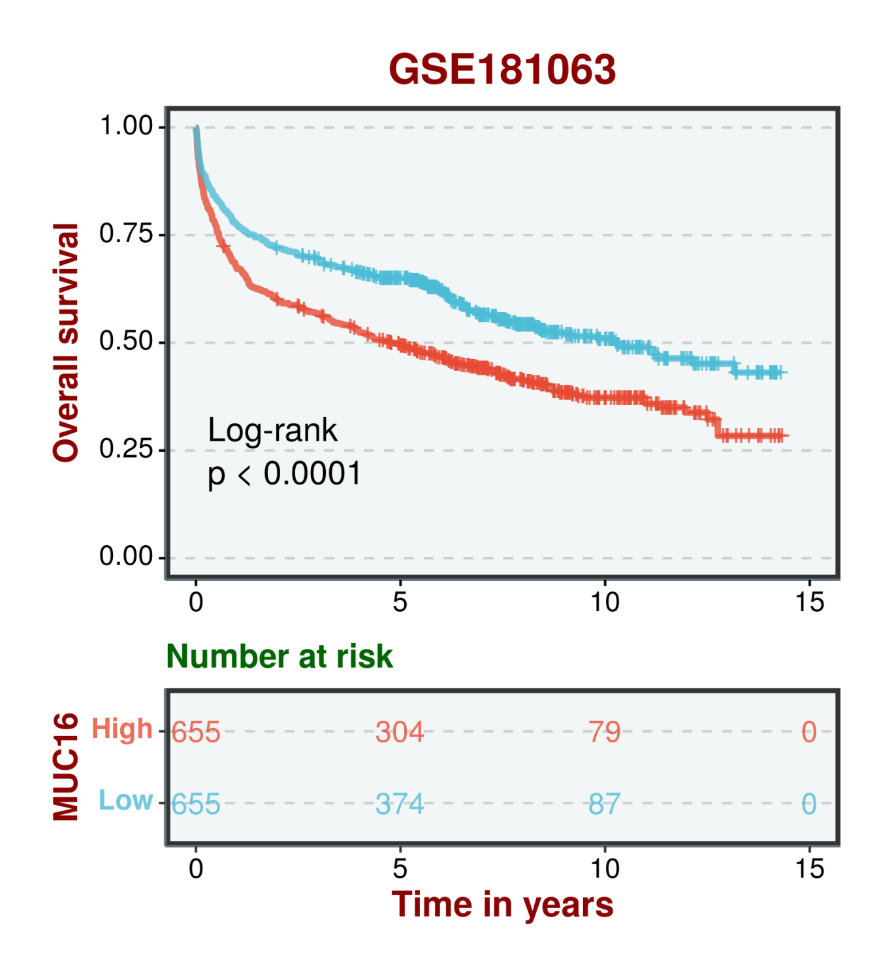


**Figure S5.** Correlation between CA125 mRNA (MUC16) levels and OS (GSE181063).
